# In‐Depth Assessment of Cytologic Features in 106 Cervical Papanicolaou Tests of Transgender Male Patients on Testosterone: An Institutional Experience

**DOI:** 10.1002/dc.70018

**Published:** 2025-09-15

**Authors:** Juanita E. Ferreira, Nikki Chiang, Xin Jing, Brian Smola, Richard L. Cantley, Judy C. Pang, Madelyn Lew

**Affiliations:** ^1^ Department of Pathology Michigan Medicine Ann Arbor Michigan USA

**Keywords:** cervical, cervical cytology, pap test, testosterone, transgender

## Abstract

**Background:**

There is a decreased cervical cancer screening rate among female to male transgender (FTMTG) patients. Data on the distinct cytologic features present in cervical cytology (CC) of those on testosterone therapy is limited.

**Methods:**

An 2017–2023 electronic database search identified CC specimens from a cohort of FTMTG patients on testosterone therapy (TT). A morphologic retrospective review of CC for cellularity and presence of key morphologic features was performed. Records were reviewed for original cytologic diagnoses and concurrent HPV test results for comparison with a cisgender female (CF) cohort.

**Results:**

106 of 132,363 (0.08%) identified CC specimens were from FTMTG patients on TT. Diagnostic rates were compared to the CF population. The most common diagnosis for both groups was “negative for intraepithelial lesion or malignancy”. The unsatisfactory rate was significantly higher in the FTMTG cohort at 21.7% (vs. 2.7%). The comparative HPV positivity rate of FTMTG and CF cohorts was 13.2% and 10.7%, respectively. Of 83 FTMTG satisfactory CC specimens, 67% showed low cellularity (narrowly meeting the adequacy threshold of 5000 well‐visualized squamous cells) and 78% showed extensive squamous atrophy. Nuclear grooves and irregular contours (features associated with transitional cell metaplasia) were observed in 18% and 23%, respectively. High N:C ratio was noted in 20% of cases.

**Conclusion:**

The higher unsatisfactory rate in FTMTG patients raises the question of whether adequacy criteria for this cohort should be adjusted. To enhance diagnostic accuracy, providing an accurate clinical history may prevent overinterpretation of features associated with transitional cell metaplasia.

## 1 Introduction

Cervical cancer incidence and mortality rates have declined by 50% from the mid‐1970s to the mid‐2000s largely due to the widespread implementation of screening tools including cervical Papanicolaou (Pap) tests and HPV co‐testing [1]. However, disparities in cervical cancer screening by gender identity persist. Gender identity is a person's internal sense of self along the gender spectrum, which may differ from their assigned sex at birth. Gender dysphoria is the distress caused by this incongruence. Transgender individuals represent a diverse population whose assigned sex at birth differs from their current gender identity or expression. Over 1.6 million adults and youth identify as transgender in the US [2]. While female‐to‐male transgender (FTMTG) patients often leverage hormone therapy to suppress or induce secondary sex characteristics, many do not undergo surgical interventions like hysterectomy. According to the National Transgender Discrimination Survey, just 21% of female‐to‐male transgender (FTMTG) individuals surveyed had undergone hysterectomy [3]. This figure highlights the continued need for cervical cancer screening.

The American Congress of Obstetricians and Gynecologists (ACOG) recommends the same screening guidelines for cisgender women and transgender men with a cervix, but the latter have lower Pap test use with 37% lower odds of being up‐to‐date with screening [4]. There are notable barriers to access to appropriate healthcare in transgender individuals, with evidence showing that they do not receive the same level of care in cervical cancer screening when compared to cisgender women [5]. Additionally, vulvovaginal atrophy secondary to testosterone therapy may make screening more challenging and painful and also can induce morphologic changes in squamous cells that impact specimen adequacy and interpretation. In fact, testosterone therapy and transgender identity are associated with up to 10‐fold higher rates of unsatisfactory Pap tests compared to cisgender women [2, 6]. Also, various cytologic changes related to hormone therapy, such as transitional cell metaplasia, may raise concern for dysplasia. However, the extent to which these findings can be generalized is unclear due to the overall paucity of literature.

Prior studies addressing these concerns have relatively limited sample sizes. Given this context, it is essential for us to collect more data to enhance our patient care for this cohort by understanding its context in terms of specimen adequacy, morphologic features, and relevant clinical correlations. Here, we present our institutional experience with cervical Pap specimens from FTMTG patients on testosterone therapy. This study aims to provide an institutional analysis of the morphologic features in these specimens as well as a comparative assessment of diagnostic rates and HPV test results with a cisgender female cohort.

## 2 Materials and Methods

We conducted a retrospective search in our pathology electronic laboratory information system (LIS) for cervical Pap specimens collected from FTMTG patients from 2017 to 2023. No vaginal Pap tests were included in our study cohort. To accurately identify the target population, a specialized database query was developed in collaboration with our department's Pathology Informatics team to retrieve cervical Pap tests from patients excluding those categorized as “female.” At our institution, FTMTG patients are classified within the system as either “male” or “unknown/not known.” The “unknown/not known” designation is most often applied when cervical cytology orders are placed by providers or during specimen accessioning, at which time gender identity information such as “transgender” or “female‐to‐male” may be recorded in the clinical documentation. This approach enabled rigorous identification and inclusion of FTMTG patients in the analytic cohort. Electronic health records were then reviewed for pertinent demographic data, information about testosterone therapy at the time of the Pap test, and any relevant gynecologic follow‐up due to resultant Pap results (i.e., cervical biopsy/excision). All Pap test results were recorded. All Pap tests were processed using the ThinPrep system (Hologic Inc., Marlborough, MA) and were evaluated with the constructs provided by the Bethesda System for Reporting Cervical Cytology (TBSRCC). Diagnostic rates for each TBSRCC category were evaluated. Additionally, any concurrent human papillomavirus (HPV) test results (Roche Cobas HPV Test (Roche Molecular Systems Inc., Pleasanton, CA)) were recorded. Diagnostic rates in cervical Pap tests and concurrent HPV results were evaluated in a comparative cohort with cisgender females from the same time period (2017–2023). A chi‐squared (*χ* ^2^) analysis was conducted to compare the results between the two groups.

Available cervical Pap ThinPrep slides from the FTMTG cohort were retrieved for a comprehensive morphologic review by a board‐certified cytopathologist (ML). Morphologic features assessed included the presence of extensive degenerative changes, nuclear grooves, prominent nucleoli, coarse chromatin, nuclear contour irregularity, high nuclear‐to‐cytoplasmic (N:C) ratios, background inflammation, extensive squamous atrophy, mitotic activity, and orangeophilia. An estimation of specimen cellularity was also provided and categorized as low (narrowly meeting the adequacy threshold of 5000 well‐visualized squamous cells), moderate (approximately 5100–10,000 well‐visualized squamous cells), or high (> 10,000 well‐visualized squamous cells). For specimens with moderate and high cellularity, an approximate estimation was made based on visual “eyeball” assessment. In cases with lower specimen cellularity, estimation was performed by counting the number of cells in 10 high‐power (40× objective) fields taken across the diameter of the slide and subsequently averaging the results.

## 3 Results

### 3.1 Diagnostic Rates and HR‐HPV Data

**FIGURE 1:**
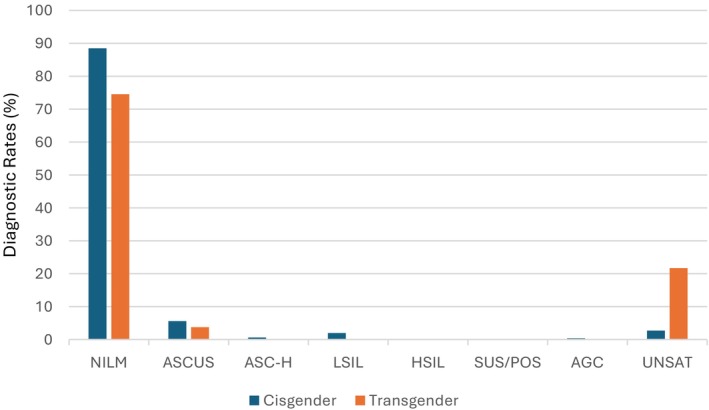
Comparison of diagnostic rates between FTMTG patients on testosterone and cisgender female patients. [Color figure can be viewed at wileyonlinelibrary.com]

We identified 132,363 total cervical cytology specimens collected between 2017 and 2023. Among these, 106 Pap tests (representing 0.08% of total) were identified from 86 FTMTG patients (aged 21–59 years) who all were undergoing testosterone treatment at the time of specimen collection. Of these 106 Pap tests, 79 (74.5%) were interpreted as negative for intraepithelial lesion or malignancy (NILM), 4 (3.8%) as atypical squamous cells of undetermined significance (ASCUS) and 23 (21.7%) as unsatisfactory for evaluation (UNSAT) (Figure 1). All cases categorized as UNSAT had a cited explanatory comment as being unsatisfactory due to insufficient squamous cellularity.

In the comparative cisgender female cohort, 116,988 (88.5%) Pap tests were interpreted as NILM. 3616 (2.7%) were UNSAT. Abnormal Pap test results in order of frequency were ASCUS (7396, 5.6%), atypical squamous cells, cannot exclude a high‐grade intraepithelial lesion (ASC‐H) (842, 0.6%), low‐grade squamous intraepithelial lesion (LSIL) (2625, 1.9%), high‐grade squamous intraepithelial lesion (HSIL) (275, 0.2%), suspicious for malignancy (29, 0.02%), and atypical glandular cells (AGC) (486, 0.4%).

For the purposes of this study, and to facilitate comparisons between cohorts, specimens were sub‐stratified to one of three diagnostic categories: NILM, “abnormal” (including all diagnoses except NILM and UNSAT), and UNSAT (Table 1). Comparison of diagnostic rates between the FTMTG and cisgender female populations demonstrated that a NILM result was the most common diagnosis in both groups, observed in 74.5% and 88.5% of cases, respectively (Figure 1). The rate of unsatisfactory (UNSAT) specimens was substantially higher in the FTMTG cohort at 21.7%, compared to 2.7% in the cisgender female cohort (Figure 1). Chi‐square (*χ* ^2^) analysis revealed that the differences in diagnostic distributions between the two cohorts were statistically significant (*p* < 0.0001).

**TABLE 1:** Comparative diagnostic rates of unsatisfactory, negative, and abnormal cases between FTMTG cohort and cisgender female cohort.

	Transgender patients	Cisgender patients
NILM	79 (74.5%)	116,988 (88.5%)
Abnormal	4 (3.8%)	11,653 (8.8%)
UNSAT	23 (21.7%)	3616 (2.7%)
Total	106	132,257

A total of 53 of the 106 FTMTG Paps (50%) had concurrent HR‐HPV testing done with a positivity rate of 13.2% (7 of 53). This was slightly higher than the cisgender female cohort, which had a positivity rate of 10.7% (9602 of 89,604) for concurrent HPV testing. Chi‐square (*χ* ^2^) analysis revealed no statistically significant differences in HR‐HPV results between the two cohorts (*p* = 0.19). Table 2 highlights the cytologic diagnosis and histologic correlates for the FTMTG HPV positive cervical cytology cases.

**TABLE 2:** Cytologic and histologic correlation in HPV‐positive FTMTG patients.

Case #	hrHPV type	Cytologic diagnosis	Histologic correlation
1	Other	NILM	Not applicable
2	Other	ASCUS	Fragments of benign endocervical mucosa
3	Other	NILM	Not applicable
4	Other	NILM	Endometrial sampling done for AUB (benign endocervical tissue and possible atrophic endometrial glands)
5	Other	ASCUS	HSIL (cervix; 2:00) and transitional cell metaplasia (cervix; 11:00)
6	16	ASCUS	Benign atrophic cervical squamous epithelium and transformation zone
7	16	UNSAT	Squamous atrophy

Abbreviations: ASCUS, atypical squamous cells of undetermined significance; AUB, abnormal uterine bleeding; HSIL, high grade squamous intraepithelial lesion; NILM, negative for intraepithelial lesion or malignancy; UNSAT, unsatisfactory.

### 3.2 Morphologic Features and Cellularity

**FIGURE 2:**
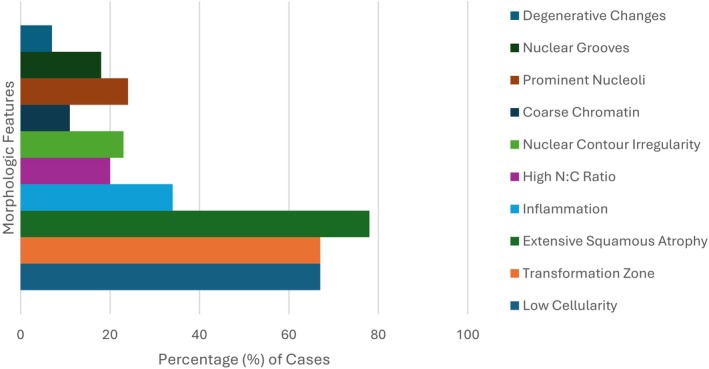
Distribution of morphologic features present in Pap tests of FTMTG patients on testosterone therapy. [Color figure can be viewed at wileyonlinelibrary.com]

**FIGURE 3:**
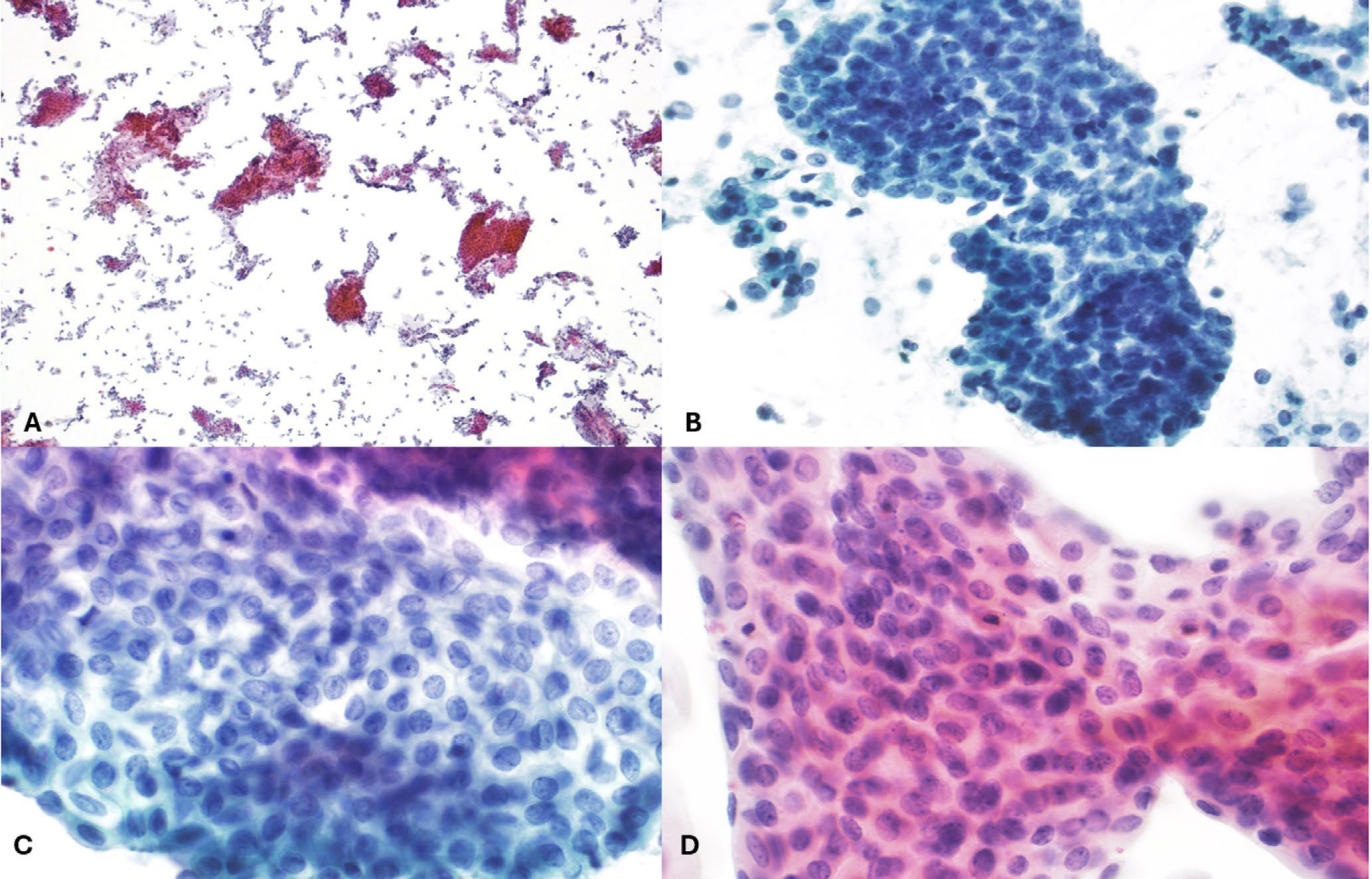
Representative ThinPrep cervical cytology images from FTMTG patients on testosterone showing epithelial atrophy (A; 4×) and high N:C ratio (B; 20×). Features suggestive of transitional cell metaplasia including characteristic longitudinal grooves (C; 60×) and overall streaming cellular appearance (D; 60×) are easily identified. [Color figure can be viewed at wileyonlinelibrary.com]

Of the 106 FTMTG Pap tests, 83 (78.3%) were originally deemed satisfactory for evaluation and subsequently assessed for cellularity as well as several morphologic features such as extensive degenerative changes, nuclear grooves, prominent nucleoli, coarse chromatin, nuclear contour irregularity, high nuclear‐to‐cytoplasmic (N:C) ratios, background inflammation, extensive squamous atrophy, mitotic activity, and orangeophilia. Of the 83 satisfactory cases, 56 (67.5%) had low cellularity (narrowly meeting the adequacy threshold of 5000 well‐visualized squamous cells), 26 (31.3%) displayed moderate cellularity (approximately 5100–10,000 well‐visualized squamous cells), and 1 (1.2%) was considered to have high cellularity (> 10,000 well visualized squamous cells). Results of the assessment for the aforementioned morphologic features are highlighted in Figure 2. The most common feature was extensive squamous atrophy, characterized by sheets of cells with scant cytoplasm, smooth nuclear contours, and a proteinaceous background (Figure 3A), observed in 78% (65 of 83) of cases. Low cellularity was also a frequent finding, noted in 67% (56 of 83) of cases. A high nuclear‐to‐cytoplasmic (N:C) ratio was present in 20% (17 of 83) of cases (Figure 3B). However, cells exhibiting a high N:C ratio specifically in small cells were rare, occurring in only 7% (6 of 83) of cases. Features suggestive of transitional cell metaplasia—including longitudinal nuclear grooves (18%; 15 of 83) and nuclear contour irregularity (23%; 19 of 83) (Figure 3C,D)—were also observed. Coarse chromatin was identified in 11% (9 of 83) of cases, prominent nucleoli in 24% (20 of 83), and extensive degenerative changes in 7% (6 of 83). The transformation zone was present in 67% (56 of 83) of cases. Mitotic figures and orangeophilia were absent in all cases.

## 4 Discussion

The aim of this study was to enhance our understanding of morphologic changes in cervical Pap tests within the cohort of FTMTG patients undergoing testosterone treatment. As noted previously, there is a relatively limited amount of information available in the current literature base [7, 8, 9, 10, 11, 12, 13, 14, 15, 16], in which study cohorts are often small and can include a variety of cervical cytology specimen preparations. While Peitzmeier et al. [6] provided a substantial cohort of 233 FTMTG patients, the focus of their study was to correlate various social and demographic patient characteristics with up‐to‐date Pap test status and did not describe morphologic features of the Pap tests. Studies that have taken a more detailed approach to morphologic evaluation of FTMTG Pap tests have limited numbers ranging from 14 to 122 patients, highlighting the need to aggregate more data sources. Additionally, some of these studies come from institutes with specialized care for transgender patients (which includes various physician subspecialists in addition to mental health clinicians who have experience working with people of diverse gender identities), underscoring the need to broaden the practice scope of the literature base [17]. Our study contributes to the literature from patient populations representative of large, tertiary care academic centers using ThinPrep to evaluate cervical Pap specimens.

### 4.1 Comparative Unsatisfactory Rates

Our study revealed an 8‐fold higher unsatisfactory rate in our FTMTG cohort (21.7%) compared to our cisgender female cohort (2.7%). Plummer et al. [16] had findings similar to those of our study in their cohort of 77 cervical Pap specimens from 71 FTMTG patients. Their study cohort had an unsatisfactory rate of 23.4% compared to 3.0% in their cisgender female atrophic control group. Lin et al. [12] also showed higher unsatisfactory rates in their FTMTG cervical Pap tests (16% vs. 2% in the comparative cisgender female group), which also leveraged ThinPrep liquid‐based cytology preparations. Torous' [11] study was composed of all SurePath cervical Pap preparations and reported a smaller difference in original unsatisfactory rates in their FTMTG cohort (3.9% vs. 0.5% cisgender female group). While Moatamed et al. [13] similarly highlighted a higher unsatisfactory rate in their study (9.8% vs. 1.6% in the cisgender female comparative group), it is noted that their study consisted of a relatively even division of SurePath and ThinPrep cervical Pap preparations. Davis et al. [14] presented an outlier in their data, with a 0% unsatisfactory rate in the FTMTG cohort, which could be at least partially attributed to the collection of samples by dedicated clinical care personnel who were trained to optimize sampling for cervical Pap tests. Their study cohort, similar to Torous, leveraged SurePath liquid‐based cytology preparations for cervical Pap tests.

Of the 83 satisfactory cases, 56 (67.5%) had low cellularity (narrowly meeting the adequacy threshold of 5000 well‐visualized squamous cells). Upon morphologic re‐review of the cases (ML), 10 cases initially diagnosed as satisfactory for evaluation were deemed borderline for adequacy when estimating cellularity by counting the number of cells in 10 high‐power (40× objective) fields taken across the diameter of the slide and subsequently averaging the results. These 10 cases were reviewed independently by 3 other board‐certified cytopathologists (JCP, RC, XJ) to assess for adequacy. All cytopathologists had a variable interpretation for adequacy, with all noting the difficulty in distinguishing between glandular cells and atrophic squamous cells. Without a provided history of collection from a FTMTG patient on testosterone, the pathologists agreed that several of these selected cases could be considered unsatisfactory. If all 10 of these cases were added to the initially reported unsatisfactory cases, our FTMTG cohort's overall unsatisfactory rate would increase significantly to 31.1%. An increase in the unsatisfactory rate upon re‐review was also reported by Torous [11], who showed an increase from 3.9% to 14%. However, Moatamed et al. [13] showed no significant change in unsatisfactory rates on re‐review. Nonetheless, there appears to be a common theme of higher unsatisfactory rates in FTMTG cohorts in prior literature findings and our study when compared to cisgender females. This raises support for lowering the adequacy threshold for these patients to 2000 squamous cells in a liquid‐based preparation, akin to recommendations from the Bethesda System for Reporting Cervical Cytology for groups of patients who have had hysterectomies, radiation therapy, or are otherwise post‐menopausal with atrophic smears.

### 4.2 Comparative Abnormal Diagnostic Category Rates

In our study, the diagnostic abnormality rate in the FTMTG cohort was lower than that of the comparative cisgender female cohort (3.8% vs. 8.8%). There were no diagnoses of LSIL, HSIL, or ASC‐H in our FTMTG cohort. Our findings are in keeping with Moatamed et al. [13], who reported that they had a lower rate of abnormal cervical Pap diagnoses in their FTMTG cohort (3.3%) than their cisgender female cohort (9.8%). However, diagnostic abnormality rates in cervical Pap tests from FTMTG cohorts in other publications were higher, ranging from 5.6% to as high as 29.2% [8, 10, 12, 13, 14, 15, 16]. In other studies that compared the diagnostic abnormality rates for a FTMTG cohort to a cisgender female comparison group [12, 15], the diagnostic abnormality rate was higher in the FTMTG cohort. In contrast to Lin et al. [12], who showed higher rates of HSIL (3% vs. 0%) in their FTMTG cohort, we did not have any HSIL diagnoses in our transgender cohort. However, this difference may be attributed to the referral of their study cohort to clinics specialized in gender‐affirming care, which may not represent the general FTMTG population or cohorts based in large urban centers. Another factor that may contribute to our relatively low diagnostic abnormality rate is the difficulty in identifying dysplastic features in the setting of atrophy.

### 4.3 Morphologic Features in FTMTG Pap Tests

As noted previously, the majority of satisfactory Pap tests (67.5%) had low cellularity, while 31.3% and 1.2% displayed moderate and high cellularity, respectively. For low cellularity cases, we used a semi‐quantitative counting method. However, cases of moderate and high cellularity were categorized using subjective “eyeball” estimation. Although this approach is commonly used in practice, we acknowledge that it lacks reproducibility and may impact the precision of categorizing cases as moderately or highly cellular. In our dataset, the impact was limited due to the very small number of high‐cellularity cases (*n* = 1). Furthermore, our study primarily focused on the distinction between low/borderline cellularity and adequate specimens, an issue previously highlighted in the literature. Studies requiring more precise delineation of cellularity may benefit from employing more objective measurement techniques, such as digital image analysis.

In our evaluation, the most common morphologic feature in cervical Pap tests deemed adequate for evaluation was extensive squamous atrophy (78% of cases), corresponding to previous reports of atrophy induced by testosterone therapy. This was also reiterated in other investigators' studies, such as in Lin et al. [12], who showed that 87% of their FTMTG cohort on testosterone also exhibited significant atrophy, and Torous [11], who found moderate to severe atrophy in 64.7% of their cases.

Other studies have noted the relatively high frequency of transitional cell metaplasia in Pap tests from FTMTG patients. As described by Weir and Bell [8], this finding is characterized by the presence of cohesive sheets with spindled nuclei displaying grooves, tapered ends, wrinkled nuclear contours, and perinuclear halos. These features can mimic dysplasia and lead to diagnostic overcalls as abnormal cervical Pap tests. In our cohort, we evaluated the presence of nuclear contour irregularity and nuclear grooves, which were observed in 23% and 18% of adequate specimens, respectively. Within the subset of cases exhibiting these nuclear features, the majority showed only rare to focal nuclear contour irregularities and grooves. The frequency of these morphologic features was lower than that of the previously reported rates of transitional cell metaplasia in cervical Pap tests, which ranged from 43.1% to 88.2% [8, 11].

Other previously reported morphologic features that may lead to diagnostic overcalls included small cells with high N:C ratios. High N:C ratios were observed in 17 of 83 adequate cases (20%), with small cells (defined as having high N:C ratios and hyperchromatic nuclei) in 7 (41.1%) of these 17 cases. Again, these findings were observed at lower frequencies compared to those reported by Torous [11], who noted that 52.9% of cases showed clusters of cells with scant cytoplasm, while 15.6% of cases showed naked, molded nuclei. Similarly, Williams et al. [8] identified small cells in 82.4% of 17 Pap smears. Acknowledging these findings as potential normal features of FTMTG Pap tests may help reduce false positive diagnoses and the resulting unnecessary treatment or patient distress. Therefore, providing clinical history relevant to FTMTG patients and details regarding testosterone therapy on cytology requisition forms may enhance diagnostic accuracy.

### 4.4 Comparative Concurrent Positive HPV Rate

Half (50%) of our FTMTG patients on testosterone treatment had concurrent high‐risk HPV testing, with a positivity rate of 13.2% (vs. 10.7% of the cisgender female cohort). Of the 7 positive HPV tests, 5 underwent subsequent histologic follow‐up, with 4 (80%) yielding benign results. In these 5 cases, the cervical Pap tests were called unsatisfactory (*n* = 1, 20%), NILM (*n* = 1, 20%), and ASCUS (*n* = 3, 60%). The remaining patient with histologic follow‐up had subsequent diagnosis of cervical HSIL with a concurrent focus of transitional cell metaplasia. Their prior cervical Pap was diagnosed as ASCUS.

Lin et al. [12] and Moatamed et al. [13] reported relatively similar HPV positivity rates (18.8% and 16%, respectively) in their FTMTG cohorts. Notably, one of the HPV‐positive Pap tests in our FTMTG cohort was deemed unsatisfactory. While Lin et al.'s positive HPV cohort had only NILM, ASCUS, and HSIL Pap diagnoses, Moatamed et al. reported that 2 of their HPV‐positive FTMTG Pap tests (16.7% of total HPV‐positive Pap tests) were also diagnosed as unsatisfactory. These findings highlight potential benefits of specimen self‐collection for primary HPV screening in this population to further optimize cancer screening and health maintenance.

## 5 Conclusion

The relatively small sample size limits the scope of our study. Although the quantity of patients prevents generalization, we believe our findings contribute to the existing pooled data and enhance statistical analysis.

Overall, our study is consistent with findings in the existing literature base in that the unsatisfactory rate of cervical Pap tests was significantly higher in our FTMTG cohort when compared to our cisgender female cohort. Additionally, cervical Pap tests from these patients frequently show extensive squamous cell atrophy and low cellularity. This raises the question of whether adequacy criteria for this cohort should be adjusted similarly to recommendations for patients who have undergone hysterectomies, radiation therapy, or have otherwise atrophic smears in the Bethesda System for Reporting Cervical Cytology. To enhance diagnostic accuracy in the evaluation of cervical Pap tests from FTMTG patients on testosterone, providing an accurate clinical history may prevent overinterpretation of features associated with transitional cell metaplasia (i.e., nuclear grooves and nuclear contour irregularity) and the presence of cells with increased N:C ratios as abnormal. Additionally, the presence of positive HPV results in the setting of unsatisfactory cervical Pap tests highlights potential benefits of alternative screening methods like self‐sample collection for primary HPV screening. Self‐sample collection may also alleviate some of the stressors associated with cervical cancer screening in FTMTG patient cohorts and thereby improve screening rates in this population.

## Author Contributions

Juanita E. Ferreira, Nikki Chiang: acquired/analyzed/interpreted data and resource materials, drafted the manuscript and contributed significant revisions on subsequent drafts, contributed to final approval of the version to be published, and agrees to be accountable for all aspects of the work in ensuring questions related to accuracy or integrity of any part of the work are appropriately investigated and resolved. Brian Smola, Xin Jing, Richard L. Cantley, Judy C. Pang: contributed revisions for intellectual content, contributed to final approval of the version to be published, and agrees to be accountable for all aspects of the work in ensuring questions related to the accuracy or integrity of any part of the work are appropriately investigated and resolved. Madelyn Lew: designed the concept of the article, acquired/analyzed/interpreted data and resource materials, drafted the manuscript and contributed significant revisions on subsequent drafts, contributed to final approval of the version to be published, and agrees to be accountable for all aspects of the work in ensuring questions related to accuracy or integrity of any part of the work are appropriately investigated and resolved.

## Conflicts of Interest

The authors declare no conflicts of interest.

## Data Availability

The data that support the findings of this study are available from the corresponding author upon reasonable request.
